# Alcohol consumption in Estonia and Finland: Finbalt survey 1994-2006

**DOI:** 10.1186/1471-2458-10-261

**Published:** 2010-05-19

**Authors:** Kersti Pärna, Kaja Rahu, Satu Helakorpi, Mare Tekkel

**Affiliations:** 1 Department of Public Health, University of Tartu, Estonia; 2 Estonian Centre of Behavioural and Health Sciences, Tallinn/Tartu, Estonia; 3 Institute for Health Development, Tallinn, Estonia; 4 National Institute for Health and Welfare, Helsinki, Finland

## Abstract

**Background:**

Alcohol consumption has been regarded as an important contributor to the high premature mortality rates. The objective of this paper was to provide an overview and comparison of alcohol consumption and its socio-demographic determinants among adults in Estonia and Finland.

**Methods:**

The study was based on a 25-64-year-old subsample of nationally representative postal cross-sectional surveys conducted in Estonia (n = 10,340) and Finland (n = 19,672) during 1994-2006. Abstinence, frequency, and the amount of alcohol consumed were examined. Logistic regression models were used to test the socio-demographic differences in alcohol consumption at least once a week. The effect of socio-demographic factors on pure alcohol consumed per week was calculated using linear regression.

**Results:**

The proportion of abstainers was 1.5 times higher among women than men in both countries. Throughout the study period, the amount of alcohol consumed per week increased for both genders in Estonia and for women in Finland, but was stable for men in Finland. In the final study year, medium risk amount of alcohol consumed per week was nearly 1.5 times higher among men in Estonia than in Finland, but about half that among women in Estonia than in Finland. Compared to ethnic majority in Estonia, alcohol consumption at least once a week was lower among men, but amount of pure alcohol drunk per week was higher among women of ethnic minority. In Finland, alcohol consumption at least once a week was more prevalent among women of ethnic minority, but the amount of pure alcohol drunk per week was lower for both gender groups of ethnic minority. Compared to married/cohabiting respondents, alcohol consumption at least once a week was less pronounced among single respondents in Finland, divorced or separated women in both countries, and widowed respondents in Estonia. Greater amount of alcohol consumed per week was more prevalent among single and divorced or separated respondents in Finland, but only among divorced or separated men in Estonia. Frequency of alcohol consumption was lower among less educated than higher educated respondents in Finland, but not in Estonia. The amount of consumed alcohol per week was higher among less educated men in Estonia, but lower among women with basic education in Finland.

**Conclusions:**

Alcohol consumption has increased in Estonia and Finland. National alcohol policies should reflect findings of alcohol epidemiology in order to introduce measures that will reduce alcohol related harm in the population effectively.

## Background

Estonia and Finland, two neighbouring countries in the Baltic Sea region, are different in certain aspects of their economic and social conditions. A former Soviet country, Estonia belongs to the group of Eastern European countries that share similar political histories and public health problems. The collapse of the Soviet Union in 1991 had enormous implications for health and for the political and economic transition during the succeeding years. Finland, however, belongs to the Nordic countries, known as well-developed welfare states characterized by a high standard of living and effective social policies [1].

Life expectancy at birth in Estonia was 60.5 for men and 72.8 for women in 1994 and thereafter started to rise. By 2006 average life expectancy was 67.4 years for men and 78.5 years for women [2]. The corresponding figures in Finland rose steadily from 73.0 for men and 80.4 for women in 1994 to 76.0 and 83.2 in 2006, respectively [3].

It is estimated that alcohol consumption is responsible for about 4% of the total disease burden in the world [4]. While regular light to moderate alcohol intake is associated with some reduction in total mortality [5,6], heavy drinking has been regarded as an important contributor to the high premature mortality rates in central and eastern Europe, particularly in the countries of the former Soviet Union [7-11]. On a national level, the severity of alcohol-related consequences depends on the frequency and volume of alcohol consumption over time and the patterns of drinking [11]. Traditional patterns in Estonia and Finland are characterized by their vodka and beer drinking cultures, non-daily drinking, irregular binge drinking episodes (e.g. during weekends and festivities), and their acceptance of public drunkenness [11]. In Finland, an additional important factor was the opening of borders (south with Estonia and east with Russia) at the beginning of the 1990s, which led to a rapid increase in tourist-imported alcoholic beverages contributing to aggregate alcohol consumption [12].

In 1994, total adult (15+ years) per capita alcohol consumption was about 8 litres per year in Estonia and Finland, and it has increased by 2004 to 16 and 10 litres, respectively [3]. Thus, there was an abrupt increase in the recorded adult alcohol consumption per capita in Estonia but not in Finland in 1994-2004. Moreover, the standardized death rate (SDR) from chronic liver disease and cirrhosis, for instance, almost doubled from 11.5 per 100,000 inhabitants in 1994 to 21.7 in 2005 in Estonia and from 9.6 to 17.6 in Finland [13]. At the same time, SDR due to accidental poisoning by alcohol was 13.4 per 100,000 in Estonia and 9.4 in Finland compared with the EU average of 0.8 in 2005.

To obtain more insight into these issues, the objective of this paper was to describe and compare the proportion of abstainers, frequency and volume of alcohol consumption and beverage preferences in Estonia and Finland, and to examine drinking habits by socio-demographic factors in both countries in 1994-2006.

## Methods

The study was based on the nationally representative cross-sectional postal surveys of the Finbalt Monitor project, which has been carried out every second year in Estonia and Finland since 1994. The national surveys were approved by the Tallinn Medical Research Ethics Committee in Estonia and the Ethical Committee of the National Public Health Institute in Finland. The target population of the Finbalt survey consisted of a simple random sample of the Estonian population aged between 16 and 64 and the Finnish population aged between 15 and 64. The samples were based on the population registries of the respective countries. In 2006 the response rate was 59% in Estonia and 65% in Finland. A detailed description of the response rates by the study year can be found elsewhere [14]. In both countries the covering letter was formulated in such a way that a respondent provided informed consent at the time of returning the questionnaire. The methodology and the questionnaires used in the surveys were harmonized to provide comparability between the participating countries [15].

This paper deals with the 25-64-years-old population from surveys carried out every even year between 1994 and 2006 in Estonia and Finland.

Alcohol consumption was measured by means of frequency and quantity questions. Frequency questions were added to the questionnaires in 2000. Usual frequency of alcohol consumption was measured by the following three questions: "How often do you usually drink spirits (1), wine (2), beer (3)?" (In 2004, time reference 'during the past 12 months' was added to the questionnaire in Estonia). Until study year 2002, the possible responses were 'never', 'a few times a year', '2-3 times a month', 'once a week', '2-3 times a week', and 'daily'. In 2004, an additional option of the response '4-6 times a week' was added to the questionnaire in Estonia. In 2006 the options 'once a week' and '2-3 times a week' were replaced by the option 'a few times a week', and the option '4-6 times a week' was removed for Estonia. In this paper, the consumption of alcohol 'once a week' is reported in the group of alcohol intake as 'a few times a week'. The respondents who answered 'never' to all three questions were defined as current abstainers. Those respondents who answered 'once a week', 'a few times a week', '2-3 times a week', '4-6 times a week', or 'daily' to any type of alcohol beverage were defined as at least weekly alcohol consumers.

The amount of alcohol consumed per week during the years 1994-2006 was estimated from the answers to the following question: "How many glasses or bottles of the following alcoholic beverages have you had during the last seven days? "(Please mark 0 if you have not had any) (1) long drinks, cider _ cans (0.33 l), (2) beer _ bottles (0.5 l in Estonia and 0.33 l in Finland), (3) wine or equivalent _ glasses (100 ml in Estonia and 120 ml in Finland), (5) strong alcohol _ shots (4 cl). In Estonia, an option concerning the consumption of weak beer was added to the questionnaire in 2004; options concerning the consumption of weak, medium, and strong beer were added in 2006. In Estonia, drinking of cider was measured in cans (330 ml), since it was regarded as belonging to the long drinks category. In Finland, the consumption of cider was measured in glasses (120 ml) and was added as an option to the questionnaire in 1998.

On the basis of this information, the usual per week intake of pure alcohol was estimated among those who had drunk at least one portion of alcohol during the previous week. The typical reported amounts of alcohol consumed were converted from litres to grams of pure alcohol per week assuming that 0.5 l of medium beer contained 20 g pure alcohol (0.33 l 12 g), 100 ml of wine 10 g, 120 ml of cider 6 g, and 4 cl of spirits 13 g (16). The following criteria for predicting the risk of per week pure alcohol consumption were used: (1) medium risk >280 g alcohol in males and >140 g in females, (2) high risk >420 g in males and >280 g in females [16].

The following socio-demographic factors were used in the analysis: age, ethnicity, marital status and education. Most of the variables analysed were self-explanatory. Age was calculated in full years (the birth year of the respondent was subtracted from the study year) and aggregated to 10-year age groups: 25-34, 35-44, 45-54 and 55-64. Ethnicity was measured by self-reported nationality (Estonian/Russian/other) in Estonia and by mother tongue (Finnish speaking/Swedish speaking/other) as revealed by information from the national population registry in Finland. Ethnicity was dichotomized as ethnic majority (Estonian in Estonia and Finnish speaking in Finland) and ethnic minority (Russian and other in Estonia, Swedish speaking and other in Finland). Marital status was categorized as married or cohabiting/single/divorced or separated/widowed. In the Estonian questionnaire, education was measured as the highest completed educational level and the total number of years. In Finland, education was measured as the total number of years. The total number of years of education was missing for the study year 2004 in Estonia; the educational system has been different for Estonians and non-Estonians but changed considerably during the study period. For this reason, the highest completed educational level was preferred for use in this study. The educational levels were divided into three categories: high (15+ years), medium (10-14 years) and low (0-9 years).

### Statistical analysis

A total of 10,340 Estonian questionnaires (4,239 men and 6,101 women) and 19,672 Finnish questionnaires (9,136 men and 10,536 women) were used in this study (Table 1). Data for men and women were analysed separately. Characteristics of the study sample, usual frequency, and volume of alcohol consumption were described by frequency tables.

**Table 1:** Number of 25-64-year-old study sample in Estonia and Finland by survey year, 1994-2006

Study year	Estonia			Finland		
	
	Men	Women	Total	Men	Women	Total
1994	474	632	1106	1340	1471	2811
1996	531	700	1231	1383	1582	2965
1998	457	630	1087	1394	1468	2862
2000	435	655	1090	1310	1553	2863
2002	420	635	1055	1230	1448	2678
2004	1039	1431	2470	1259	1525	2784
2006	883	1418	2301	1220	1489	2709

Total	4239	6101	10340	9136	10536	19672

Frequency of alcohol consumption in 2000-2006 was analysed on the basis of 6,916 Estonian (2,777 men and 4,139 women) and 11,034 Finnish (5,019 men and 6,015 women) questionnaires. Questionnaires with missing information concerning frequency of alcohol intake (1,005 in Estonia and 264 in Finland) were excluded.

Associations between the frequency of alcohol consumption and socio-demographic variables were estimated using logistic regression models. Consumption of any type of alcohol at least once a week (compared to less than once a week) was included in the model as a dependent variable; the study year and the socio-demographic characteristics (age group, ethnicity, marital status, and education) served as explanatory variables. The results of crude and fully adjusted logistic regression analysis were presented as prevalence odds ratios (POR) with 95% confidence intervals (CI). Questionnaires that lacked information about the frequency of alcohol consumption and/or socio-demographic factors (1,114 in Estonia and 471 in Finland) were excluded before logistic regression analysis.

Mean, standard deviation (SD), and median of pure alcohol consumption per week (in grams) in 1994-2006 were calculated for respondents who had drunk at least one portion of alcohol during the previous week. Questionnaires with missing or zero amounts of alcohol consumption (3,904 in Estonia and 5,595 in Finland) were excluded.

The effect of socio-demographic variables and the study year on the amount of pure alcohol consumption were assessed using linear regression models. The amount of pure alcohol consumption per week was included in the model as a dependent variable; the study year and the socio-demographic characteristics (age group, ethnicity, marital status and education) served as explanatory variables. The results of crude and fully adjusted linear regression models were presented as differences in the amount of pure alcohol consumption (in grams) with 95% confidence intervals. The p-value for the linear trend between the amount of pure alcohol and the study year was calculated. Questionnaires that lacked information about socio-demographic factors (99 in Estonia and 234 in Finland) were excluded before linear regression analysis.

## Results

Table 2 shows the socio-demographic characteristics of the sample. About three quarters of the respondents in both countries were married or cohabiting. Ethnic minority (mainly Russians) constituted nearly one third of the sample in Estonia. Only a few respondents represented ethnic minority in Finland. The proportion of respondents with a higher education was lower in Estonia than in Finland.

**Table 2:** Characteristics of respondents in Estonia and Finland, 1994-2006

	Estonia				Finland			
	
Characteristic	Men		Women		Men		Women	
	
	n	%	n	%	n	%	n	%
Age group								
25-34	1072	25.3	1439	23.6	1920	21.0	2421	23.0
35-44	1136	26.8	1502	24.6	2345	25.7	2716	25.8
45-54	1061	25.0	1614	26.5	2678	29.3	2990	28.4
55-64	970	22.9	1546	25.3	2193	24.0	2409	22.9
Marital status								
married/cohabiting	3232	76.2	4046	66.3	6756	73.9	7813	74.2
single	553	13.0	626	10.3	1556	17.0	1327	12.6
divorced/separated	364	8.6	901	14.8	722	7.9	1064	10.1
widowed	65	1.5	500	8.2	69	0.8	295	2.8
unknown	25	0.6	28	0.5	33	0.4	37	0.4
Ethnicity								
ethnic majority	2951	69.6	4143	67.9	8587	94.0	9943	94.4
ethnic minority	1254	29.6	1924	31.5	549	6.0	593	5.6
unknown	34	0.8	34	0.6	0	-	0	-
Education								
higher	712	16.8	1410	23.1	2427	26.6	3577	34.0
secondary	2655	62.6	3829	62.8	4075	44.6	4601	43.7
basic or less	845	19.9	808	13.2	2473	27.1	2188	20.8
unknown	27	0.6	54	0.9	161	1.8	170	1.6

Total	4239	100	6101	100	9136	100	10536	100

### Abstinence and frequency of alcohol consumption

**Figure 1:**
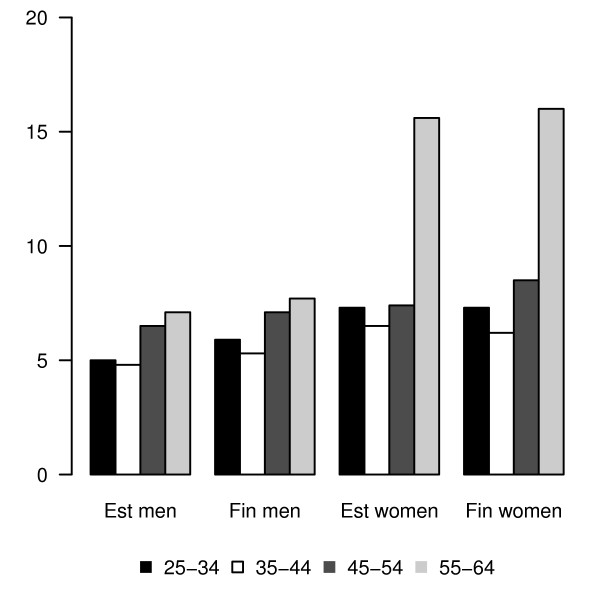
**Proportion (%) of current abstainers by gender, country and age group, Finbalt survey 2000-2006**. Overall, women in Estonia and Finland were about 1.5 times as likely as men to be current non-drinkers of alcohol (5.8% and 6.6% of men, 9.2% and 9.5% of women, respectively) in 2000-2006. The proportion of abstainers by gender was similar in both countries. There were no age differences between male abstainers in Estonia and Finland. Among women, the highest proportion of abstainers was in the oldest age group in both countries (15.6% in Estonia and 16.0% in Finland).

Overall, women in Estonia and Finland were about 1.5 times as likely as men to be current abstainers (5.8% and 6.6% of men, 9.2% and 9.5% of women, respectively) in 2000-2006. There were no age differences between male abstainers in Estonia and Finland. Among women, the highest proportion of abstainers was in the oldest age group in both countries (15.6% in Estonia and 16.0% in Finland) (Figure 1).

Table 3 shows the frequency of consumption of different types of alcohol. Among the respondents in Estonia and Finland, the most popular alcohol beverage for at least weekly drinking was beer (52.2% and 55.2% for men, 10.7% and 22.1% for women, respectively), followed by spirits among men (22.4% and 20.6%, respectively) and wine among women (9.8% and 18.2%, respectively). There were more daily beer drinkers, but fewer weekly beer drinkers among men in Estonia than in Finland. No difference was found between the two countries among men who drank spirits at least once a week, but almost twice as many Finnish men as Estonian men drank wine at least weekly. Nearly twice as many women drank beer or wine at least once a week in Finland as in Estonia.

**Table 3:** Frequency (%) of drinking each type of beverage by gender, country and age group, 2000-2006

Type of beverage	Estonia					Finland				
	
	25-34	35-44	45-54	55-64	Total	25-34	35-44	45-54	55-64	Total
**Men**										
Spirits										
never	9.3	7.3	9.1	11.3	9.2	10.1	10.0	11.2	10.5	10.5
a few times a year	33.6	30.8	30.3	31.9	31.7	45.6	44.6	40.1	36.1	41.3
a few times a month	40.6	36.9	36.0	32.7	36.7	32.6	28.9	25.5	25.3	27.7
a few times a week	15.6	23.0	21.8	21.4	20.4	11.4	15.9	22.1	26.5	19.6
daily	0.9	1.9	2.7	2.8	2.0	0.3	0.6	1.1	1.7	1.0
Beer										
never	11.3	9.5	15.2	18.1	13.3	12.7	9.8	11.6	12.7	11.6
a few times a year	11.4	11.7	11.7	13.9	12.1	11.8	12.9	15.6	17.8	14.8
a few times a month	25.7	21.2	20.1	22.0	22.3	23.0	19.6	15.9	16.6	18.4
a few times a week	40.5	43.5	38.2	32.5	38.9	50.1	52.1	49.7	44.4	49.0
daily	11.1	14.1	14.7	13.5	13.3	2.5	5.5	7.2	8.5	6.2
Wine										
never	21.5	23.6	26.8	32.4	25.7	25.1	19.5	23.5	21.9	22.4
a few times a year	43.5	40.2	39.7	36.8	40.3	40.1	41.2	37.5	38.4	39.2
a few times a month	27.6	23.0	21.3	19.8	23.1	23.0	22.1	18.0	15.6	19.4
a few times a week	7.2	12.3	10.8	10.0	10.0	11.5	16.7	19.2	22.3	17.9
daily	0.2	0.8	1.4	1.0	0.8	0.3	0.5	1.7	1.8	1.2
**Women**										
Spirits										
never	30.5	25.9	21.7	30.7	27.1	20.8	22.2	23.6	32.3	24.7
a few times a year	47.6	47.3	52.3	51.7	49.7	63.6	61.1	56.0	49.7	57.5
a few times a month	17.6	21.5	20.6	14.8	18.6	13.6	12.5	14.2	11.9	13.1
a few times a week	4.3	5.0	5.3	2.7	4.3	1.8	4.1	6.1	5.8	4.6
daily	0.1	0.4	0.1	0.2	0.2	0.1	0.1	-	0.3	0.1
Beer										
never	43.2	43.0	47.3	60.8	48.4	44.4	31.5	31.8	44.0	37.4
a few times a year	25.7	26.5	26.4	22.8	25.4	21.9	24.3	26.6	26.3	24.9
a few times a months	20.1	16.3	15.3	10.3	15.6	18.4	17.5	16.0	10.6	15.6
a few times a week	10.3	13.1	10.2	5.6	9.9	14.9	26.1	23.6	18.0	21.0
daily	0.7	1.1	0.9	0.5	0.8	0.4	0.6	2.0	1.1	1.1
Wine										
never	11.5	13.4	18.4	28.9	18.0	18.7	15.5	19.5	23.8	19.4
a few times a year	40.6	39.8	45.5	45.5	42.9	41.5	40.3	39.8	40.5	40.5
a few times a month	36.8	33.0	27.3	20.1	29.4	27.3	24.2	20.4	16.5	22.0
a few times a week	10.7	13.0	8.4	5.0	9.3	12.4	19.2	19.2	17.7	17.4
daily	0.4	0.7	0.4	0.4	0.5	0.2	0.7	1.0	1.4	0.8

**Figure 2:**
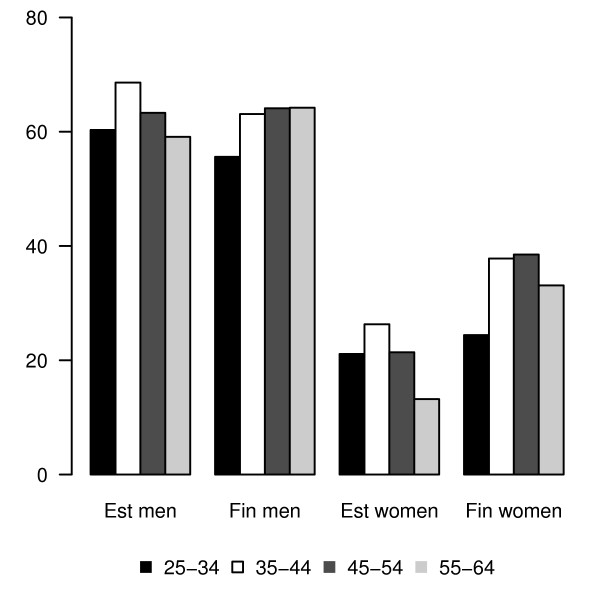
**Proportion (%) of respondents consuming alcohol at least once a week by gender, country and age group, Finbalt survey 2000-2006**. Consumption of any type of alcoholic beverages at least weekly was similar among men in both countries (62.9% and 62.3%, respectively), but it was lower among women in Estonia than in Finland (20.7% and 33.9%, respectively). Approximately three times as many men as women in Estonia and twice as many in Finland drank alcohol at least once a week. The proportion of people who drank any type of alcoholic beverages at least once a week varied across age groups. It was the highest in the age group 35-44 among men and women in Estonia but grew steadily with age among men in Finland, and reached a higher level among 35-54-year-old women in Finland.

Consumption of any type of alcoholic beverage at least weekly was similar among men in both countries (62.9% and 62.3%, respectively), but it was 1.6 times lower among women in Estonia than in Finland (20.7% and 33.9%, respectively). The proportion of people who drank any type of alcoholic beverage at least once a week varied across age groups; it was the highest among 35-44-year-old men and women in Estonia, but increased steadily with age among men in Finland, and reached the highest level among 45-54 year-old women in Finland (Figure 2).

The association between socio-demographic factors and frequency of alcohol drinking at least once a week was explored (Table 4). Compared to the first study year, weekly alcohol drinking was slightly higher among men in Estonia and Finland in 2004, but lower among men in Estonia in 2006. Compared to the youngest age group, alcohol consumption at least once a week was higher among men in all older age groups in Finland, but only among 35-44-year-old men in Estonia. The probability of drinking alcohol at least once a week was lower for ethnic minority men compared with similar individuals in ethnic majority in Estonia. No relationship between the frequency of alcohol consumption and ethnicity was established among men in Finland. In comparison with married/cohabiting men, drinking alcohol weekly or more frequently was lower among widowed men in Estonia and among single men in Finland. Frequency of alcohol drinking was lower among men with a basic education than those with higher education in Finland, but not in Estonia.

**Table 4:** Prevalence odds ratios (POR) and 95% confidence intervals (CI) for consuming any type of alcoholic beverage at least once a week by gender and country, 2000-2006

Characteristic	Estonia		Finland	
	
	Crude POR	Adjusted POR*	Crude POR	Adjusted POR*
**Men**				
Study year				
2000	1	1	1	1
2002	0.81 (0.61-1.08)	0.75 (0.56-1.00)	1.04 (0.89-1.23)	1.02 (0.87-1.21)
2004	1.41 (1.09-1.82)	1.39 (1.08-1.80)	1.26 (1.07-1.49)	1.21 (1.03-1.43)
2006	0.66 (0.52-0.85)	0.64 (0.50-0.83)	1.01 (0.86-1.19)	0.95 (0.80-1.12)
Age group				
25-34	1	1	1	1
35-44	1.40 (1.11-1.77)	1.44 (1.13-1.83)	1.34 (1.13-1.60)	1.34 (1.12-1.60)
45-54	1.11 (0.89-1.40)	1.20 (0.94-1.53)	1.42 (1.20-1.68)	1.54 (1.29-1.85)
55-64	0.96 (0.76-1.21)	1.02 (0.79-1.32)	1.41 (1.19-1.69)	1.62 (1.33-1.98)
Ethnicity				
ethnic majority	1	1	1	1
ethnic minority	0.56 (0.47-0.67)	0.53 (0.44-0.63)	1.02 (0.80-1.30)	1.01 (0.78-1.29)
Marital status				
married/cohabiting	1	1	1	1
single	0.95 (0.74-1.21)	0.95 (0.73-1.23)	0.63 (0.54-0.73)	0.70 (0.60-0.82)
divorced/separated	1.06 (0.80-1.42)	1.00 (0.74-1.35)	0.96 (0.78-1.19)	0.97 (0.78-1.20)
widowed	0.45 (0.22-0.91)	0.41 (0.20-0.84)	1.24 (0.54-2.86)	1.20 (0.51-2.78)
Education				
higher	1	1	1	1
secondary	1.10 (0.89-1.36)	1.08 (0.87-1.35)	0.98 (0.85-1.12)	0.92 (0.80-1.06)
basic or less	0.99 (0.76-1.29)	0.97 (0.73-1.29)	0.66 (0.56-0.78)	0.56 (0.47-0.67)
**Women**				
Study year				
2000	1	1	1	1
2002	0.62 (0.47-0.82)	0.60 (0.45-0.80)	1.02 (0.87-1.19)	1.02 (0.87-1.20)
2004	1.11 (0.89-1.40)	1.07 (0.84-1.35)	1.14 (0.98-1.33)	1.13 (0.97-1.32)
2006	0.48 (0.38-0.62)	0.46 (0.36-0.59)	1.03 (0.88-1.20)	0.98 (0.84-1.15)
Age group				
25-34	1	1	1	1
35-44	1.34 (1.07-1.68)	1.38 (1.10-1.74)	1.90 (1.61-2.24)	2.03 (1.72-2.41)
45-54	1.02 (0.81-1.28)	1.06 (0.83-1.34)	1.96 (1.67-2.30)	2.30 (1.94-2.73)
55-64	0.56 (0.43-0.74)	0.60 (0.45-0.80)	1.55 (1.31-1.83)	2.01 (1.67-2.42)
Ethnicity				
ethnic majority	1	1	1	1
ethnic minority	0.92 (0.76-1.11)	0.87 (0.72-1.07)	1.46 (1.16-1.84)	1.43 (1.13-1.82)
Marital status				
married/cohabiting	1	1	1	1
single	0.89 (0.68-1.16)	0.85 (0.65-1.12)	0.69 (0.58-0.82)	0.74 (0.61-0.88)
divorced/separated	0.76 (0.59-0.98)	0.77 (0.60-1.00)	0.85 (0.70-1.02)	0.79 (0.66-0.96)
widowed	0.51 (0.34-0.76)	0.65 (0.43-0.99)	0.74 (0.50-1.10)	0.73 (0.49-1.08)
Education				
higher	1	1	1	1
secondary	0.83 (0.69-1.00)	0.81 (0.67-0.98)	0.78 (0.69-0.88)	0.67 (0.60-0.76)
basic or less	0.65 (0.46-0.90)	0.77 (0.55-1.09)	0.62 (0.53-0.73)	0.50 (0.42-0.60)

*Each POR was adjusted for all other characteristics in the table

Alcohol consumption at least once a week was lower among women in Estonia in 2002 and 2006 than in the first study year (Table 4). Compared to the youngest age group, frequency of alcohol drinking was higher among women in all older age groups in Finland, but only among 35-44 year-old women in Estonia. Frequency of alcohol drinking was the lowest among women in the oldest age group in Estonia. Alcohol consumption at least once a week was more prevalent in ethnic minority women compared to the women in ethnic majority in Finland, but not in Estonia. Compared to married/cohabiting women, weekly alcohol consumption was slightly lower among divorced or separated women in both countries, among widowed women in Estonia and single women in Finland. Frequency of alcohol drinking was lower among women with secondary and basic education than higher education in both countries. After adjustment the association with a basic education appeared not to be significant among women in Estonia.

### Amount of pure alcohol consumed during previous week

Table 5 shows the amount of pure alcohol drunk, the mean and median of pure alcohol consumed per week and the percentage of respondents with medium and high risks of drinking. Among men in Estonia, the median of pure alcohol consumed per week increased throughout the study period. The proportion of men with medium or high risk alcohol intake followed the same trend. Among men in Finland, the median of pure alcohol consumption and medium risk alcohol drinking were stable, but the proportion of high risk alcohol drinkers per week increased slightly throughout the study period. Compared to Finland, the proportion of men with medium and high risk alcohol consumption per week was about the same in 1994, but nearly 1.5 times higher in 2006 in Estonia.

**Table 5:** Mean and median pure alcohol consumption (g) during the previous week and the proportion of medium and high risk drinkers (respondents who had drunk at least one portion of alcohol) by gender, country and study year, 1994-2006

Study year	Estonia					Finland				
	
	n	Mean (SD) g/week	Median g/week	Medium risk* %	High risk** %	n	Mean (SD) g/week	Median g/week	Medium risk* %	High risk** %
**Men**										
1994	362	128 (147)	79	9.1	3.9	1047	136 (126)	102	10.9	3.7
1996	363	112 (110)	78	7.4	1.9	1043	131 (128)	98	9.5	3.8
1998	329	123 (155)	80	7.9	2.7	1131	143 (137)	105	12.0	3.7
2000	329	147 (185)	100	9.7	6.1	1030	137 (141)	99	10.0	4.6
2002	310	144 (157)	97	11.3	4.5	970	139 (176)	97	9.6	3.6
2004	795	164 (169)	113	14.8	6.4	1006	143 (132)	110	10.7	4.0
2006	686	165 (192)	105	16.2	7.0	931	145 (146)	100	11.6	5.2
**Women**										
1994	300	43 (67)	26	4.3	1.0	915	57 (54)	38	6.9	1.0
1996	334	35 (35)	26	2.7	-	996	62 (62)	48	7.5	1.2
1998	337	39 (38)	26	2.4	0.3	1008	63 (59)	48	9.3	1.1
2000	368	43 (45)	30	3.5	0.8	993	59 (66)	38	7.5	1.1
2002	340	42 (84)	25	2.6	1.2	953	60 (64)	38	7.8	1.5
2004	794	46 (54)	30	4.4	1.4	1059	64 (64)	48	9.5	1.1
2006	789	49 (73)	30	4.7	1.0	995	67 (77)	48	10.0	1.7

* >280 g pure alcohol per week in men and >140 g in women

** >420 g pure alcohol per week in men and >280 g in women

Median of pure alcohol consumed and medium risk alcohol drinking per week were lower among Estonian than Finnish women in 1994-2006 (Table 5). During the study period, the proportion of medium risk weekly alcohol drinkers increased among women in both countries. The proportion of women in Estonia whose amount of alcohol consumed per week was medium risk in 2006 was less than half that in Finland. The proportion of high risk drinking women remained stable throughout the study period in both countries.

The effect of the study year and socio-demographic factors on the amount of pure alcohol consumed per week was explored (Table 6). The years 1994-2006 showed an increasing trend in the amount of pure alcohol consumed per week among men in Estonia (p < 0.001) but not in Finland (p = 0.065). The amount of pure alcohol consumed per week was the lowest in the oldest age group among men in both countries. In Finland only, the amount of pure alcohol consumed weekly was lower in ethnic minority men. Compared to married/cohabiting men, pure alcohol consumed during the week was higher among divorced or separated men in both countries, but higher among single men in Finland only. Significantly more pure alcohol was consumed per week by less educated men in Estonia, but not in Finland.

**Table 6:** The effect of study year and socio-demographic factors on pure alcohol consumption (g) during the previous week (respondents who had drunk at least one portion of alcohol) by country and gender, 1994-2006

Characteristic	Estonia				Finland			
	
	Crude estimate		Adjusted estimate*		Crude estimate		Adjusted estimate*	
	
	β	(95% CI)	β	(95% CI)	β	(95% CI)	β	(95% CI)
**Men**								
Study year								
1994	ref		ref		ref		ref	
1996	-16	(-40, 8)	-13	(-37, 12)	-6	(-18, 6)	-6	(-18, 6)
1998	-4	(-29, 21)	-2	(-26, 23)	7	(-5, 18)	6	(-6, 18)
2000	22	(-3, 47)	27	(2, 52)	1	(-11, 13)	0	(-12, 12)
2002	16	(-9, 42)	18	(-7, 43)	2	(-10, 15)	2	(-10, 14)
2004	36	(15, 57)	38	(17, 59)	7	(-5, 20)	8	(-5, 20)
2006	37	(16, 58)	43	(22, 64)	8	(-5, 21)	7	(-5, 20)
p-value for linear trend		<0.001		<0.001		0.054		0.065
Age group								
25-34	ref		ref		ref		ref	
35-44	0	(-15, 16)	3	(-13, 19)	0	(-9, 10)	3	(-7, 13)
45-54	-5	(-22, 11)	-6	(-22, 11)	4	(-5, 14)	10	(0, 20)
55-64	-42	(-60, -25)	-44	(-62, -25)	-22	(-32, -12)	-14	(-25, -3)
Ethnicity								
ethnic majority	ref		ref		ref		ref	
ethnic minority	-7	(-20, 6)	-5	(-18, 7)	-35	(-48, -21)	-31	(-44, -17)
Marital status								
married/cohabiting	ref		ref		ref		ref	
single	24	(6, 42)	13	(-6, 31)	38	(29, 48)	36	(27, 46)
divorced/separated	45	(24, 66)	43	(23, 64)	61	(49, 73)	61	(48, 73)
widowed	-46	(-98, 6)	-28	(-80, 24)	12	(-26, 50)	23	(-15, 61)
Education								
higher	ref		ref		ref		ref	
secondary	21	(6, 37)	18	(3, 33)	5	(-3, 13)	3	(-4, 11)
basic or less	16	(-3, 36)	26	(7, 46)	-11	(-20, -2)	-9	(-19, 1)
**Women**								
Study year								
1994	ref		ref		ref		ref	
1996	-8	(-18, 1)	-8	(-17, 2)	5	(-1, 11)	5	(-1, 11)
1998	-4	(-14, 5)	-4	(-14, 5)	6	(0, 12)	6	(0, 12)
2000	0	(-9, 9)	1	(-8, 10)	2	(-4, 8)	2	(-4, 8)
2002	-1	(-11, 8)	0	(-9, 10)	1	(-4, 7)	1	(-5, 6)
2004	3	(-6, 11)	4	(-5, 12)	7	(1, 12)	7	(1, 12)
2006	6	(-2, 14)	7	(-1, 15)	10	(4, 16)	10	(4, 15)
p-value for linear trend		0.001		<0.001		0.010		0.015
Age group								
25-34	ref		ref		ref		ref	
35-44	-1	(-6, 5)	-1	(-6, 5)	7	(3, 11)	8	(4, 13)
45-54	-1	(-7, 5)	-2	(-8, 4)	6	(2, 10)	9	(4, 13)
55-64	-6	(-13, 0)	-8	(-14, -1)	-6	(-11, -2)	-3	(-8, 2)
Ethnicity								
ethnic majority	ref		ref		ref		ref	
ethnic minority	6	(1, 10)	6	(2, 11)	-8	(-14, -1)	-8	(-14, -1)
Marital status								
married/cohabiting	ref		ref		ref		ref	
single	3	(-4, 10)	4	(-3, 11)	12	(7, 17)	12	(8, 17)
divorced/separated	1	(-5, 7)	1	(-5, 7)	9	(4, 14)	9	(4, 14)
widowed	-1	(-9, 8)	0	(-9, 9)	-2	(-11, 8)	3	(-7, 13)
Education								
higher	ref		ref		ref		ref	
secondary	1	(-3, 6)	1	(-3, 6)	-1	(-5, 2)	-1	(-5, 2)
basic or less	6	(-2, 14)	9	(0, 17)	-9	(-14, -5)	-7	(-12, -2)

*Each estimate was adjusted for all other characteristics in the table.

The whole study period revealed an increasing trend in the amount of pure alcohol consumed per week among women in Estonia (p < 0.001) and Finland (p = 0.015) (Table 6). Compared to the women in the youngest age group, the amount of pure alcohol consumed per week was lower in the oldest age group in Estonia, but higher among 35-44 and 45-54-year-old women in Finland. The amount of pure alcohol consumed per week was higher among women of ethnic minority rather than women from ethnic majority in Estonia. This association was reversed in Finland. Pure alcohol consumption per week was higher among single and divorced or separated women than married/cohabiting women in Finland and lower among women with basic education than those with higher education.

## Discussion

The present article focused on the results of alcohol consumption among 25–64-year-old respondents of Finbalt Monitor surveys carried out every even year in Estonia and Finland during the period 1994-2006.

### Limitations

Before discussing the results, one has to consider the limitations of the survey. All questionnaire surveys of alcohol consumption are somewhat problematic because of inaccurate categorization of alcohol intake. People usually underestimate their consumption. In addition, heavy drinkers are generally less likely to participate in the surveys. Therefore the findings of the study need to be interpreted with caution. It is also important to consider that the degree of underreporting may vary among countries even if similar measurement techniques and standardized questionnaires are used. In this study, the questions concerning alcohol consumption varied slightly by study year and country. For example, questions about the frequency of alcohol consumption were formulated "How often do you drink spirits/wine/beer?" in Estonia in 2000 and 2002, "How often did you usually drink spirits/wine/beer during the last 12 month?" in Estonia in 2004 and 2006, and "How often do you usually drink spirits/wine/beer?" in Finland. Thus, caution must be exercised in making cross-country comparisons [17]. However, a major limitation was that because the survey was designed to obtain data on health behaviour in the adult population rather than alcohol consumption, the questions about drinking were basic and included no distinction between lifelong abstainers, former drinkers, or any of the standard instruments to detect hazardous drinking (excessive drunkenness, hangover, going to bed with one's clothes on because of drunkenness). Absence of data on the pattern of problem drinking means that the present study could underestimate the harmful drinking. Furthermore, the volume of alcohol consumed might have been underestimated because the figure was based on the question about alcohol consumption during the previous week. It may have excluded a rather significant proportion of less regular medium or high risk drinkers. Unfortunately, the common question "Have you consumed any alcohol during the previous year?" was not mandatory in the Finbalt survey questionnaire and was missing from the Estonian questionnaire. Therefore the frequency of drinking taking into account only the alcohol beverage most frequently consumed, was underestimated. Finally, the overall sample of the Finbalt survey was relatively large, but the small size of the samples per study year and the small size of certain sub-groups reduced the power to detect significant differences. For this reason pooled analysis rather than trend analysis was used to describe the association between alcohol consumption and socio-demographic factors. Despite these caveats, several inferences can be drawn.

### Abstinence and frequency of alcohol consumption

A first crude indicator of alcohol consumption behaviour is the proportion of abstainers. The overall proportion of non-drinkers by gender did not differ between the countries. Moreover, Rehn et al [18] reported that the eight countries with the lowest proportions of abstainers are all northern European countries (including Estonia and Finland). In the present study, the proportion of abstainers was, not surprisingly, 1.5 times higher among women in both countries. This confirms the results of previous studies in Finland and Estonia [19]. Also, the NORBALT survey (1999) reported, that the non-drinking rate was much lower in men than in women (10% and 23%, respectively) in Estonia [20]. Thus, it seems that in Estonia the gender gap has decreased during the past ten years.

The proportions of abstainers by gender differed considerably between the countries, and this might be explained by their different traditions with respect to alcohol. For example, the gender gap has been small in countries such as Norway, Denmark, and Germany, but Sweden, the Netherlands and the United Kingdom revealed larger gender differences in the proportion of abstainers [19].

Overall, the preferred alcohol beverages were similar in Estonia and Finland, but the frequency of drinking each type of beverage and at least weekly consumption of any type of alcohol differed between the countries. There were very few daily drinkers in either country regardless of beverage or gender. The only exception to this pattern was beer drinking among men in both countries, but especially in Estonia. This could be explained by different attitudes towards life style and health in Estonia and Finland. Also, previous studies have shown that daily drinking was rather rare in the Baltic countries and Finland [10,19]. In addition, McKee et al [10] reported that the only group in which drinking was at all common was Estonian male beer drinkers (13%). Countries can often be categorized as mainly beer, wine or spirits countries. The results of this study confirm that Estonia and Finland are primarily beer-drinking countries. They both used to be spirits-drinking countries. In Finland, the proportion consuming spirits was the highest until the 1990s [21]. Simpura et al [12] reported that the era of spirits-drinking as a dominant feature in the Scandinavian countries had already ended by the 1960s. In Estonia, spirits were the most popular type of alcohol beverages as recently as 1999. At the same time, the consumption of beer doubled among men in Estonia during 1994-1999 [20]. Nevertheless, spirits drinking did not disappear in these countries; only its relative position became weaker. The present study reported that more than a fifth of men in Estonia and Finland were at least weekly drinkers of spirits. As in to the Commonwealth of Independent States, this finding supports the assumption that the consumption of strong spirits is still common in Estonia [22]. While spirits drinking was low among women in both countries, the drinking of beer and wine at least weekly was more common among women in Finland. This could explain the higher prevalence of alcohol consumption among women in Finland.

Consumption of any type of alcohol beverage at least weekly was similar among men in Estonia and Finland, but it was much lower among women in Estonia than in Finland. Previous Estonian studies showed the same proportion of weekly alcohol drinkers [19]. The lower proportion of alcohol consumption at least once a week among women in Estonia could be explained by the fact that during the Soviet period alcohol consumption among women was low.

### Amount of alcohol consumed

Throughout the study period, the proportion of medium risk drinkers increased among men in Estonia and among women in Finland. Prevalence of medium risk drinkers among women in Estonia decreased after the first study year, but increased during the succeeding years. In the first study year the proportion of medium and high risk drinkers among men was lower in Estonia than in Finland, but the final study year revealed the opposite. Prevalence of medium risk alcohol drinkers was lower among women in Estonia than those in Finland throughout the study period. According to the HFA database [3], there is some supporting evidence for this.

### Socio-demographic differences in the frequency of alcohol consumption and amount consumed

In general, the socio-demographic factors influenced the frequency and amount of alcohol consumed differently among men and women in the two countries. No clearcut trend was found in the frequency of alcohol consumption in Estonia and Finland during 2000-2006. The lower frequency of alcohol consumption in Estonia in the final study year might be explained by the replacement of the option 'once a week' with 'a few times a week' in the question about alcohol consumption frequency in the Estonian questionnaire in that year. Thus, some respondents who consumed alcohol once a week could answer that they had consumed alcohol only a few times a month in 2006. At the same time, the whole study period 1994-2006 showed an increasing trend in the amount of pure alcohol consumed by men in Estonia and by women in both countries. One reason for increasing trend in alcohol consumption could be liberal alcohol policy in both countries. Moreover, previous studies in Finland reported that in the long-term, alcohol consumption of women has increased relatively more than of men in Finland, even if drinking is still more common among men [23,24].

Compared to the youngest age group, frequency of alcohol consumption was more prevalent among both genders in all older age groups in Finland and among 35–44-year-olds in Estonia, but lower among the 55-64-year-old women in Estonia only. The lower frequency of alcohol drinking in the youngest age group could be explained by the irregularity of alcohol consumption among young respondents. The amount of pure alcohol consumed per week was higher among Finnish women in the age groups 35-44 and 45-54 than among the youngest age group. At the same time, compared to the youngest age group, the amount of consumed pure alcohol per week was lower in the oldest age group among men in both countries and among women in Estonia.

The increase of alcohol drinking among women over 35-year-old follows a longerterm trend in Finland, reflects more liberal attitudes towards alcohol consumption and the egalitarian position of women in society [23,24]. At the same time, low alcohol consumption among 55-64-year-old women in Estonia could be explained with traditionally low alcohol drinking habits among women during the Soviet period.

Alcohol consumption weekly or more frequently was lower among men, but the amount of pure alcohol consumed per week was higher among women of ethnic minority in Estonia. At the same time, McKee et al [10] found that weekly alcohol drinking was lower among non-Estonian men and women in Estonia in 1997. The inconsistency in the findings concerning the frequency of alcohol consumption among women might be explained by a time difference in these studies (they were conducted in different years). However, compared to ethnic majority in Finland, alcohol drinking at least once a week was more common among women, but the amount of pure alcohol consumed per week was lower among both genders of ethnic minority.

Frequency of alcohol consumption was slightly less pronounced among divorced or separated women in both countries and widowed respondents in Estonia. Single respondents drank less frequently but consumed more alcohol per week in Finland. Divorced or separated men in both countries, but women in Finland only showed similar drinking patterns in the amount of alcohol consumed per week. A previous report of the Finbalt study [14] also indicated that heavy drinking was more common among non-married people.

The present study showed that less educated respondents in Finland and women in Estonia drunk alcohol with lower frequency. The fact that McKee et al [10] found no association between education and weekly alcohol consumption in Estonia in 1997 could again be explained by the time difference in these studies. A greater weekly consumption of pure alcohol was associated with lower education among men in Estonia only. One could speculate that better educated adults drink alcohol more frequently than less educated ones but consume less alcohol per week. Also, a survey conducted in Izhevsk in Russia reported that lower education was strongly associated with hazardous drinking in working age men [25]. Nevertheless, women with a basic education consumed less pure alcohol per week than more highly educated women in Finland. This confirms the finding of Helasoja et al [14] that heavy drinking was more common among better educated adults in Finland, which is a typical drinking pattern in Northern Europe [26].

## Conclusion

Although Estonia and Finland are neighbouring countries, they differed markedly in the frequency and amount of drinking, and in the association between alcohol consumption and socio-demographic factors. Alcohol consumption has increased in Estonia and Finland indicating that alcohol should be a concern for public health in this region. National alcohol policies should reflect the findings of alcohol epidemiology in order to reduce alcohol-related harm in the population effectively.

## Competing interests

The authors declare that they have no competing interests.

## Authors' contributions

KP: made a substantial contribution to the conception and the design of the study, interpretation of the data, drafted the manuscript and has been involved in revising the manuscript critically. KR: participated in the design of the study, performed statistical analyses, has been involved in the interpretation of the data and in revising the manuscript critically. SH: has taken part in collecting the Finnish data and preparing the Finnish data set and designing the study, was involved in the interpretation of the data, and critically revised the manuscript. MT: principal investigator of the Finbalt study in Estonia was involved in the interpretation of the data and critically revised the manuscript. All authors read and approved the final manuscript.

## Pre-publication history

The pre-publication history for this paper can be accessed here:

http://www.biomedcentral.com/1471-2458/10/261/prepub
